# Sun protection to improve vaccine effectiveness in children in a high ambient ultraviolet radiation and rural environment: an intervention study

**DOI:** 10.1186/s12889-016-3966-0

**Published:** 2017-01-06

**Authors:** Caradee Y. Wright, Patricia N. Albers, Angela Mathee, Zamantimande Kunene, Catherine D’Este, Ashwin Swaminathan, Robyn M. Lucas

**Affiliations:** 1 Environment and Health Research Unit, South African Medical Research Council, 1 Soutpansberg Road, Pretoria, 0001 South Africa; 2 Department of Geography, Geoinformatics and Meteorology, University of Pretoria, Pretoria, South Africa; 3 Environment and Health Research Unit, South African Medical Research Council, Johannesburg, South Africa; 4 University of Johannesburg, Johannesburg, South Africa; 5 National Centre for Epidemiology and Population Health Research School of Population Health, College of Medicine, Biology and Environment, The Australian National University, Canberra, Australia; 6 The Canberra Hospital, Canberra, Australia; 7 National Centre for Epidemiology and Population Health, Research School of Population Health, The Australian National University, Canberra, Australia

**Keywords:** Immunisation, Vaccination, Sun exposure, Measles, Intervention study, Africa

## Abstract

**Background:**

Vaccination is a mainstay of preventive healthcare, reducing the incidence of serious childhood infections. Ecological studies have demonstrated an inverse association between markers of high ambient ultraviolet (UV) radiation exposure (e.g., sunny season, low latitude of residence) and reduction in the vaccination-associated immune response. Higher sun exposure on the day prior to and spanning the day of vaccination has been associated with a reduced antigen-specific immune response independent of skin pigmentation. The South African Department of Health’s Expanded Programme on Immunisation provides free vaccinations in government primary health care clinics. In some areas, these clinics may have only a small waiting room and patients wait outside in full sun conditions. In rural areas, patients may walk several kilometres to and from the clinic. We hypothesised that providing sun protection advice and equipment to mothers of children (from 18 months) who were waiting to be vaccinated would result in a more robust immune response for those vaccinated.

**Methods:**

We conducted an intervention study among 100 children receiving the booster measles vaccination. We randomised clinics to receive (or not) sun protection advice and equipment. At each clinic we recorded basic demographic data on the child and mother/carer participants, their sun exposure patterns, and the acceptability and uptake of the provided sun protection. At 3–4 weeks post-vaccination, we measured measles IgG levels in all children.

**Discussion:**

This is the first intervention study to assess the effect of sun protection measures on vaccine effectiveness in a rural, real-world setting. The novel design and rural setting of the study can contribute much needed evidence to better understand sun exposure and protection, as well as factors determining vaccine effectiveness in rural Africa, and inform the design of immunisation programmes. (TRN PACTCR201611001881114, 24 November 2016, retrospective registration)

## Background

Vaccination is an important tool to prevent common infections and is a mainstay of preventive healthcare. Childhood vaccination programmes have reduced the infectious disease burden in many countries, but require effective vaccines and high vaccination coverage. In South Africa, where a large proportion of the population has reduced immunity to infection due to the high prevalence of HIV/AIDS and childhood malnutrition [1], effective vaccination against infectious diseases is particularly important.

To develop effective immune protection, a vaccine must induce a robust and long lasting antigen-specific immune response in the host. A number of factors are known to influence vaccine effectiveness including host factors (e.g., age, gender, genetic makeup, immunosuppressive state or medications) and vaccine factors (e.g., dose, route of administration, formulation, cold chain integrity) [2]. However, there is a growing body of research that suggests that environmental exposures – specifically exposure of the skin to ultraviolet (UV) radiation – may modulate immune responses in ways that could reduce vaccine effectiveness [3].

UV radiation has been shown to suppress antigen-specific immune processes in humans and animals, via direct and indirect (e.g., via cutaneous production of vitamin D) mechanisms. Exposure of cells within the epidermis and dermis to UV radiation sets off a complex signalling cascade of soluble immune mediators that modulate immune cell interactions within the skin and draining lymph node. The result is local and systemic immunosuppression that is antigen-specific in nature. The adaptive immune response (rather than the innate immune response) is specifically suppressed following UV irradiation of the skin [4, 5] or eyes [6].

Animal studies have shown that UV irradiation before or after vaccination results in a reduction in the delayed type hypersensitivity (DTH) response, increased microbial load or more severe clinical symptoms when the animals were challenged with the live organism or agent, compared to non-irradiated animals [7–10]. There are only a few relevant studies in humans and these have mainly used proxies of exposure to UV radiation.

Early studies demonstrated a relationship between lower seroconversion following vaccination [11] and summer season (compared to winter) [12], higher temperatures in tropical areas versus temperate areas [13] and being closer to the Equator (low latitude) versus being further away [14–16].

There has been only one published experimental trial involving healthy (adult) human volunteers that examined the effects of exposure to UV radiation on vaccination [17]. Healthy volunteers received whole-body UV irradiation (UVB) on five consecutive days; an equal number of un-irradiated individuals acted as their controls. All participants were vaccinated on day 6 with recombinant hepatitis B surface antigen, which was repeated after 1 and 6 months. Results showed natural killer cell activity and contact hypersensitivity were suppressed in UV-irradiated participants. Conversely, no differences between the groups were observed in the hepatitis B specific T cell response or antibody response. Norval and Woods [18] suggest that lack of effect on the T-cell specific and antibody response could be explained by the use of aluminium hydroxide as an adjuvant in the vaccine. This excites a strong Th2 response, whereas UV irradiation primarily suppresses Th1 immune responses. The authors also noted that the high dose of the vaccine could have overwhelmed any UV-induced immune suppression [19]. Later, genetic analyses showed that there was UV-induced immune suppression of antigen-specific antibody production in UV-irradiated participants who carried a minor allelic variant of the *IL-1ß* gene [17]. Another study showed that exposure to UV radiation resulted in a shift toward a stronger Th2 response, away from a Th1 response; this would result in a less effective response to later exposure to measles or poliovirus infections [20]. Most recently, we have shown that, in young adults, personal exposure to higher levels of solar UV radiation on the day prior to vaccination, and in the peri-vaccination period, was associated with a significant decrease in the antigen-specific cell-mediated immune response to a novel antigen, keyhole limpet haemocyanin [21]. There was no evidence that darker skin pigmentation altered the immune suppressive effect of exposure to UV radiation. This is consistent with past studies [22, 23]. The effect of solar UV radiation exposure on the immune response to diseases on the childhood vaccination schedule has not been tested, but could be significant in regions with high ambient UV radiation.

### Measles in South Africa

One infectious disease that is epidemic prone in South Africa, but preventable through vaccination, is measles. The measles virus is active in the mucus of the nose and throat. After sneezing and coughing, droplets are expelled to surfaces and remain infectious for about 2 hours. Measles is characterized by a red blotchy rash, red eyes, high temperature and a dry cough. The disease mainly affects young children and remains a leading cause of infant mortality, including in South Africa [24, 25].

The measles vaccine uses a live attenuated form of the measles virus. The Centre for Disease Control recommends children receive two doses of the measles vaccine, the first between the ages of 12 and 15 months and the second between the ages of 4 and 6 years [26]. Vaccination earlier than 12 months has been shown to result in significantly lower seroconversion rates because of persistent maternal IgG antibodies [27]. Maternal transfer of IgG antibodies has been demonstrably lower among children of mothers with high HIV-1 viral load during the third trimester of pregnancy [28].

In South Africa, nearly 30% of measles cases occur in infants under 12 months of age. The National Department of Health’s Expanded Programme on Immunisation (implemented in 1995) recommends that the first measles vaccine is administered at 9 months and the second dose at 18 months. From 1996 to 2004, first dose coverage of measles vaccine remained constant (76 –83%), while second dose coverage was slightly lower in some places (63 –78%). From 1999 to 2002, there were less than 60 cases of measles reported annually and no associated deaths [29].

The Department of Health administers vaccines in government primary health care clinics for free. In some areas, these clinics may have only a small waiting room and patients wait outdoors under full sun conditions. Use of sun protection among Africans is not common practice except, for example, among Africans with oculocutaneous albinism (OCA). In rural areas, patients may walk several kilometres to and from the clinic. This means that children may have quite high levels of sun exposure in the days leading up to, on the day, and in the days immediately following, vaccination.

It is clearly important to ensure that children are vaccinated and have a strong immune response that leads to protection. In light of the recent evidence showing that higher levels of sun exposure in the period leading up to and immediately following vaccination is associated with a demonstrable decrease in antigen-specific immune response, we hypothesised that providing sun protection advice and equipment to mothers of children who are waiting for measles vaccinations will result in an enhanced immune response. This in turn should provide improved protection against infection for those vaccinated.

### Study aims and objectives

The study aimed to test whether providing sun protection advice and equipment to mothers of children (from 18 months) waiting for vaccination with the booster measles vaccine will result in an improved immune response to the vaccination. The study objectives were to: (1) test the uptake and acceptability of sun protection advice and shade equipment, by clinic patients at the intervention clinic; (2) assess recruitment, consent, and follow-up (including barriers to these); and (3) compare the level of measles-specific antibodies in the intervention and control clinic patients 3–4 weeks post-vaccination. Results of this study will provide an understanding of the acceptability and realistic uptake of the intervention at the health clinics, and the use by clients of the sun protection advice and equipment provided as well as post-vaccination antibody levels in the intervention and control arms.

## Methods

### Setting

**Fig. 1:**
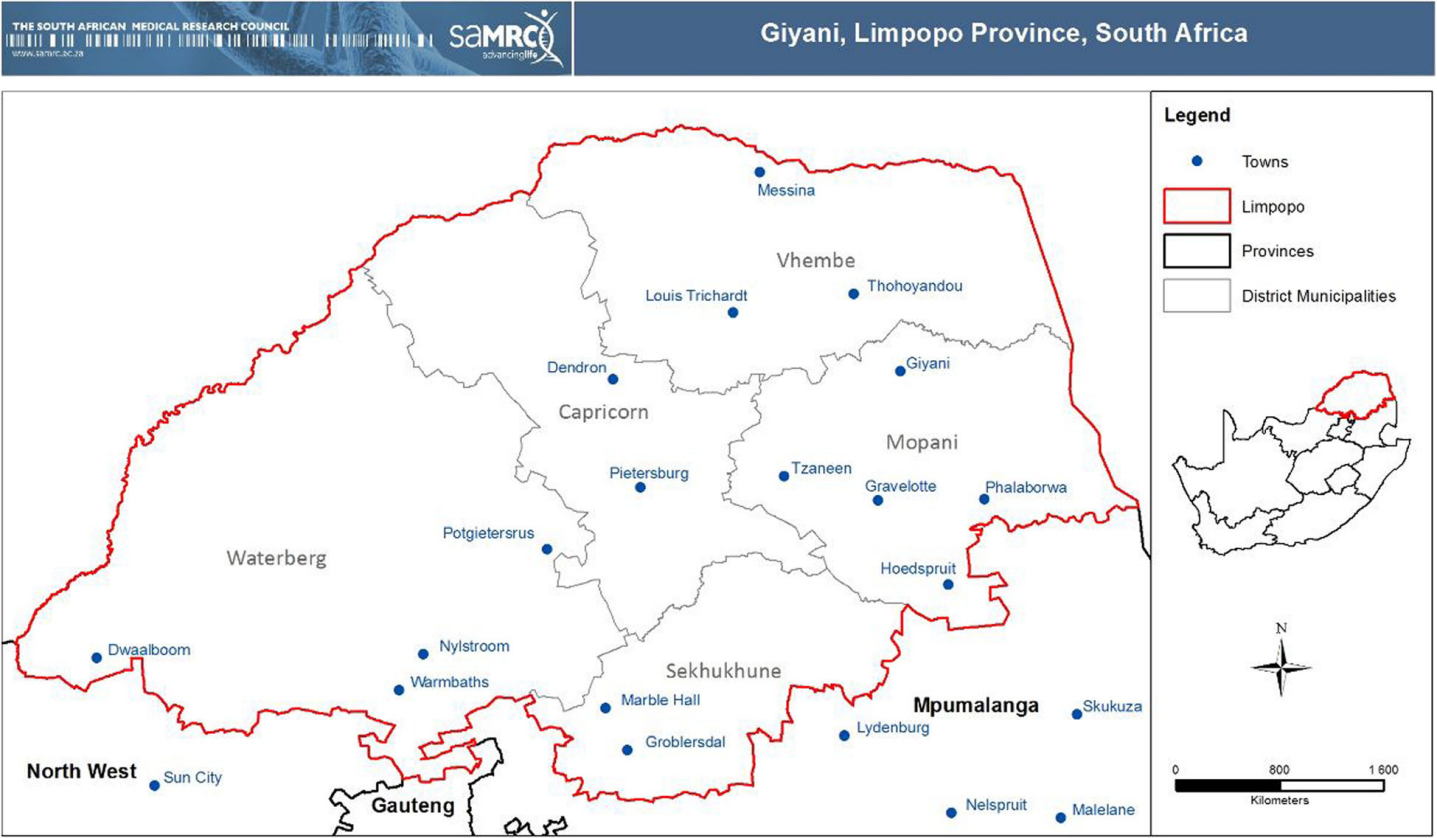
Location of Mopani District Municipality and Giyani in Limpopo Province, South Africa (Map produced by Thandi Kapwata, South African Medical Research Council)

The study was conducted in primary health care clinics in the Greater Giyani Local Municipality, Limpopo Province (see Fig. 1). Limpopo province has communities living with among the highest levels of poverty in the country [30]. In a nationwide survey assessing inequalities to health care, it was noted that nearly 20% of household respondents from Limpopo delayed seeking health care as a result of transport costs. Long queues also accounted for delayed care-seeking [31].

### Ambient solar UV environment

**Fig. 2:**
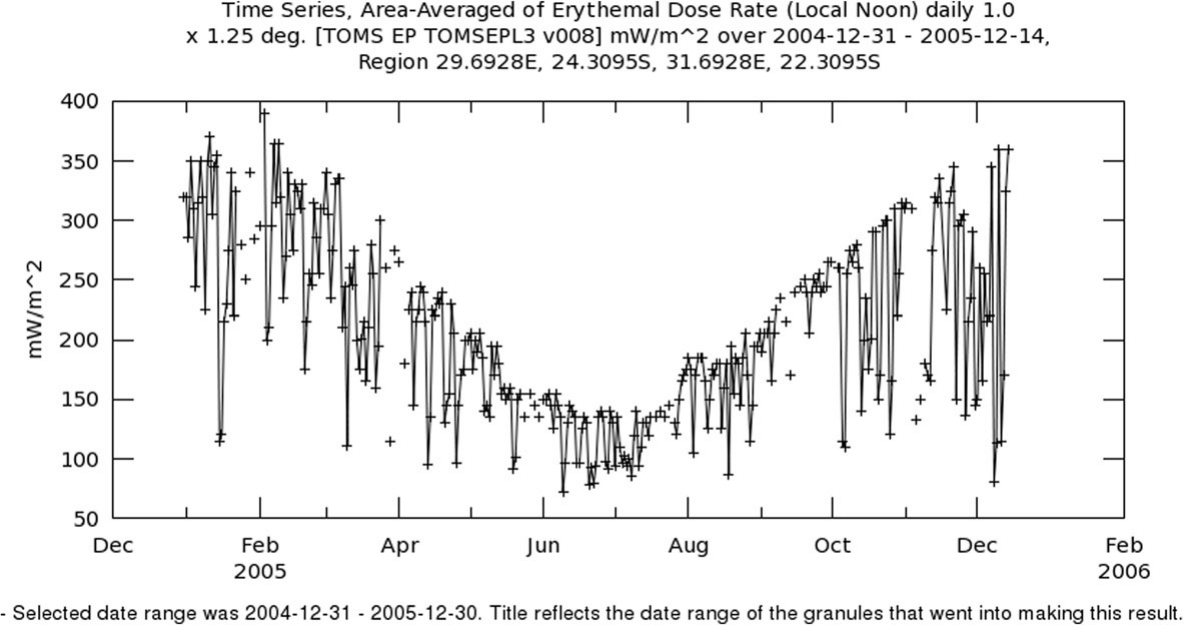
Annual variation in ambient solar UV radiation at Giyani, Limpopo Province. The data were extracted from the Ozone Monitoring Instrument (OMI) on the Aura Satellite which is part of the National Aeronautics and Space Administration (NASA) A-train satellite constellation. The data was obtained from Giovanni online data system, developed and maintained by the NASA Goddard Earth Science Data and Information Services Centre

Ambient solar UV radiation levels are relatively high during the austral summer, spring and autumn (Fig. 2) in this region of Limpopo Province. Summertime, cloud-free, midday solar erythemal UV radiation levels (UVB, wavelength range from 280 to 320 nm) in Giyani range from ~ 250 to 350 mWm^−2^. This converts to an Ultraviolet Index (UVI) range of ~ 10 to 14 (maximum for the day) during the summer months [32].

### Community engagement

Permission to carry out the study was obtained from the Limpopo Provincial Department of Health. The study team met with government officials and district nurses to discuss the study plan and visit several primary health care clinics. The study employed research nurses who were recruited from the study area and were fluent in English as well as the local languages, namely Tsonga, Northern Sotho and Sotho. The research nurses were the primary connection to the community members when they visited each primary health care clinic. In addition to completion of study tasks, the research nurses provided each mother/guardian enrolled in the study with a flyer about the study as well as other relevant Department of Health information on the importance of vaccination. At the completion of the study, the research nurses will be working with the study investigators to provide feedback and results to the study participants, clinics and Limpopo Department of Health.

### Selection of study sites

Officials from the Limpopo Provincial Department of Health provided the study team with the names and locations of all of the clinics in Greater Giyani. Several clinics were visited to establish their suitability as study sites, in terms of waiting room size, capability to provide space for research nurses. Suitable sites were similar in regard to size of the service community, demographic and economic factors of the community, training levels of the clinic staff, and having limited indoor waiting space. In addition, the study sites needed to be more than 25 km apart in order to minimise participants changing between the two clinics during the course of the study. The eligible sites were shortlisted and 2 sites were randomly allocated to be the ‘intervention’ or ‘no intervention’ site.

### Eligibility, consent and confidentiality

The study population included children aged 18 months or older, presenting at the selected clinics for their second measles vaccination. Eligibility criteria included that the child had received the first measles vaccine, their mother or guardian was deemed able to comprehend the research and complete the sun exposure diary, and was capable of signing consent for the child to be enrolled in the study, the mother had a copy of the child’s Road to Health Chart, and the mother confirmed that they would be available for the duration of the study (4 weeks). Informed consent was obtained from mothers 18 years or older with children of 18 months or older.

All study documents were developed in English and translated for use by the research nurses and the participants. The research nurse read the information sheet and consent form with the mother in the mother’s home language and answered any questions the mother had. Unique identifier codes were allocated to each study participant. Data from the various collection tools were combined using the unique identifier.

### Approach and data collection

**Fig. 3:**
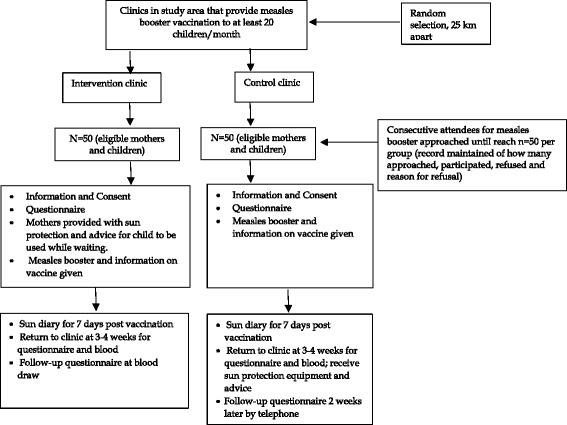
Study and recruitment process

The study and recruitment process is outlined in Fig. 3. Mothers of children aged 18 months and older, arriving to have the measles vaccination at either of the participating clinics, were invited by the research nurse to participate in the study. The reason for participating provided to the mothers was that they would receive information on whether the measles vaccine administered to their child is working as it should be. Informed consent was signed by mothers who agreed to their child’s participation. Data were collected from the Road-to-Health Chart and a questionnaire completed by the mother, with assistance from the research nurse if necessary (see below).

At the intervention clinic, the mothers of the enrolled children were provided with sun protection advice and equipment, including a hat, a long sleeved top, an umbrella and effective sunscreen. The mother was asked to apply the sun protection equipment to the child while waiting for vaccination and to continue using it for 1 week following the vaccination, whenever the child was outdoors. Mothers in both groups were asked to record sun protection practices and equipment used by the children during the first week following vaccination in a pre-prepared, simple diary. Children at control and intervention sites then received the measles vaccination according to the Health Department protocol (including routine height and weight measurements) and using the standard vaccine preparation method, and were asked to return to the clinic 3–4 weeks later for blood testing. The date and time of administration of the measles vaccine for each participant was noted by the research nurse, since this may affect immune response [33].

As mothers were travelling long distance to come to the clinic, after the blood-taking visit they were given travel cost reimbursement of R100. Following the blood draw visit, the mothers in the intervention group completed a brief questionnaire on their acceptance and use of the sun protection equipment and participants at the control site received the sun protection equipment. Control group mothers/guardians were telephoned 2 weeks after the blood-taking visit to telephonically complete the questionnaire on their acceptance and use of the sun protection equipment. Questions included ‘what did you like about using the sun protection equipment on your child’; ‘was it easy to use the sun protection equipment’; ‘did your child like wearing/using the sun protection equipment we provided to you’; and ‘any further or additional comments’. Open-ended questions were written in local language and translated (and back translated for quality control). Each clinic received gazebos for shade (the intervention clinic at the start of the study and the control clinic at the end of the study) so that mothers who were sitting outside because the waiting room was full were provided with sun protection.

### Road to health data

Data collected from the child’s Road-to-Health Chart included sex, date of birth, birth weight, birth length, birth head circumference, problems during pregnancy/birth/neonatally, APGAR score, gestational age and details related to the first measles vaccination (e.g., date received, batch number, weight and height at time of vaccination and previous vaccination record).

### Baseline questionnaire

The questionnaire was based on that used in an Australian study [34] and tailored for local conditions. Key components of the questionnaire were 1) general questions, including socio-demographic, health, skin and sun exposure questions, about mother and/or child (including breastfeeding, height and weight and current medication); 2) details of the travel to and from the clinic, e.g., time taken, route, shade; 3) duration of waiting at the clinic; 4) usual use of sun protection on the child; and 5) child’s usual time spent outside. The questionnaire also sought information on the HIV status of the child as this may impair the immune response to vaccination [35].

### Child sun exposure diary

Child sun exposure and protective behaviours were assessed using a pre-prepared child sun exposure diary, adapted from one previously used by this group [36]. The diaries are picture-based and simple to use. A parent or guardian records the child’s main whereabouts (indoors or outdoors) for the morning, noon and afternoon periods of the day. They also record what, if any, sun protective equipment was used. The diaries were completed for 7 days following the second measles vaccination, and returned to the research nurse at the blood-taking visit post-vaccination.

### Blood collection and IgG antibody levels

At the blood-taking visit, the registered research nurse drew 2 ml of blood into a serum separator tube after first applying an EMLA local anaesthetic patch, from both intervention and control participants. Serum was tested for measles IgG antibody levels at the National Institute for Communicable Diseases Centre for Vaccines and Immunology laboratory, using the Enzygnost® Anti-Measles Virus/IgG ELISA kit (Siemens, Germany). The blood samples were received in several batches. On receipt, the serum was separated from the clot by centrifugation, transferred to labelled tubes and stored at −20 °C until all the study samples were collected and received. All study samples were tested on the same day. Testing was performed according to the manufacturer’s protocol and the optical density (OD) readings were converted to quantitative titres of measles IgG (in units of mIU/mL) using the α-method calculation as described in the kit insert: log (mIU/mL) = α x corrected ΔOD^β^ where α and β are lot-specific values.

To obtain the actual titre of measles IgG, the anti-log of the value obtained was calculated. This ELISA kit interprets specimens with OD < 0.1 as negative, 0.1 < OD > 0.2 as equivocal (indeterminate) and OD > 0.2 as positive which after conversion to a quantitative measure gives roughly <150 mIU/mL as negative, 150–350 mIU/mL as equivocal (indeterminate) and >350 mIU/mL as positive. An individual is considered to be IgG-positive if OD > 0.2, i.e., IgG > 350 mIU/mL. The estimated threshold for clinical protection is 120 mIU/mL using the plaque-reduction neutralization (PRN) test which measures only functional antibodies involved in virus neutralization, but since this test is labor-intensive it is generally not used for serosurveys which usually have a very large sample size. The ELISA kit used in this study measures all anti-measles antibodies of IgG class, not just neutralizing antibodies. In general, PRN and ELISA tests compare well, except at very low antibody levels.

### Data management and analysis

All data were entered into a structured database. Recruitment, consent and follow-up rates (Aim 1) will be reported overall and by group, and compared between sites using the chi-squared test (including barriers to these. Compliance with and acceptability of sun protection measures at the clinic will be presented for the intervention site only and reported use of sun protection in the weeks post-intervention reported for both sites separately and overall, using frequencies and proportions or means, with 95% Confidence intervals (CIs). To assess measles antibody levels, we will examine the distribution of antibody levels, obtain estimates of the antibody levels in each of intervention and control groups, difference in antibody level between groups, and variance and 95% confidence intervals for these parameters (or medians and quartiles as appropriate).

We will compare the antibody levels (in geometric mean titres) between the control and intervention groups using the *t*-test or non-parametric equivalent if the antibody levels are not normally distributed. We will use multiple linear regression to examine the factors associated with levels of measles antibodies (across all children tested), including age, sex, body mass index (weight and height), HIV status, sun exposure, sun protection coverage and clinic attended (i.e., intervention versus control group).

### Sample size

There are no relevant previous studies on which to base a sample size calculation. We plan to recruit 100 patients in total, 50 in each of the intervention and control clinics. This number will allow estimation of recruitment rates within clinics with 95% Confidence Intervals (CI) within ± 10% and within clinic estimation of outcomes with 95% CI within ± 14% for proportions and ± 0.3 standard deviations for continuous measures, and differences between groups of 28% for binary measures and 0.6 standard deviations for continuous measures in univariable and regression analyses.

## Discussion

This randomised controlled trial is the first of its kind in rural southern Africa. Other studies have considered sun protection practices among children, for example those with OCA [37], but never before has sun protection been considered in relation to vaccine effectiveness in a human setting, in rural Africa. Given the geographical setting for the study, and the rural nature of the communities and clinics, to ensure smooth implementation of the proposed study, the following factors and issues needed to be considered and addressed at the study clinics: (1) vaccine stock shortages are common in South Africa; hence we had to ensure that there was sufficient stock at the clinics during the duration of the study; and (2) incorrect transport and storage of vaccines can impair their effectiveness; hence we ensured that the clinics had a fully operating fridge set at the correct temperature for storage of the vaccines.

Vaccination rates, considering all vaccines, in South Africa have been relatively poor, with a recent estimate by the World Health Organization of approximately 79% and for measles second vaccine of about 82% [38]. There have been several recent outbreaks of measles. In Limpopo, coverage for 1^st^ measles vaccination in 2011 was 100% and for the measles booster, it was 92% [39]. However, even a small reduction in the effectiveness of the vaccination in producing a protective antibody response could have important implications for the risk of measles infection in this population.

We acknowledge the limitations of this study. In particular, the use of sun protection equipment and completion of the child sun exposure diaries will rely on mothers or guardians. Research nurses will provide the rationale for the use of the sun protection equipment and advice on its correct use. Questionnaire and diary data will be self-reported and thus subject to recall bias. Research nurses check the diaries on their return and resolve any missing data with the parent or guardian. However, we have used a strong study design – the first randomised controlled trial of sun protection to improve vaccine effectiveness in a childhood population. The setting is one in which the opportunity for high sun exposure is considerable, and the risks of low effectiveness vaccination high. The study will also provide an indication of the potential for public awareness advice about simple steps that can improve vaccine effectiveness to reduce the burden of infectious diseases and improve immunity.

## Abbreviations

DTH: Delayed type hypersensitivity
IgG: Immunoglobulin
OCA: Oculocutaneous albinism
OD: Optical density
UVA: Ultraviolet radiation-A
UVB: Ultraviolet radiation-B
UVR: Ultraviolet radiation
